# Recent conclusions regarding the reconstructive microsurgery of peripheral nerves

**Published:** 2008-04-15

**Authors:** Dumitrescu-Ionescu Doina

**Affiliations:** * Head of Plastic and Reconstructive Microsurgery Clinic University Hospital, Bucharest

**Keywords:** reconstructiv microsurgery, peripheral nerve lesion, amputation

## Abstract

The introducing of reconstructive microsurgery has meant not only the addition of microsurgical microscopes and instruments, but a change, a progress towards a new concept, the concept of the microsurgical reconstruction of tissues. The microscope and the instruments themselves are only a means of utilizing this new concept to good effect since the mere use of the microscope and of the instruments according to the old concept of tissue reconstruction cannot be considered to be reconstructive microsurgery.

From December 1979 through to December 2005, more than 3.000 patients with peripheral nerve lesions were operated on by the same microsurgeon, the author Doina Ionescu-Dumitrescu.

The conclusions are based on the following:

• A huge amount of work involved in carrying out microsurgical reconstructions of over 7,500 peripheral nerves in over 3,000 patients, 1,800 of which were nerve transplants for defects of peripheral nerves of the extremities, for posttraumatic brachial plexus paralyses (91), for replantations and/or revascularizations following partial or complete amputations of the extremities (24 out of which 23 successful) or for free transfers of functional composite tissues (53). For a more accurate picture of such an effort one should consider the operation time that these types of reconstruction involve: between 3 and 12 hours for each patient under general anaesthesia and for both the anaesthetist and the microsurgeon.

• Experimental microsurgery on rabbit ears

• The results of the histopathological examination of 500 postoperative neuromas of peripheral nerves repaired traditionally

• The Moberg test

• Pre, intra and postoperative monthly observations of the patients until their full recovery according to the criteria set by the International Reconstructive Microsurgery Society (postoperative intervals of 6-12-24 months)

• Taking pictures and recording pre, intra and postoperative stages

• The patients’ professional, social and familial reintegration

• The patients’ state of mind; level of cooperation

• Comparing results with those of classic and palliative repairs

• Comparing the data resulting from this experience with the information provided by the specialist literature of the world

• Completing the internationally defined reconstructive procedures with the personal ones, to produce a new concept

The introducing of reconstructive microsurgery has meant not only the addition of microsurgical microscopes and instruments, but a change, a progress towards a new concept, the concept of the microsurgical reconstruction of tissues. The microscope and the instruments themselves are only a means of utilizing this new concept to good effect since the mere use of the microscope and of the instruments according to the old concept of tissue reconstruction cannot be considered to be reconstructive microsurgery.

From December 1979 through to December 2005, more than 3.000 patients with peripheral nerve lesions were operated on by the same microsurgeon, the author Doina Ionescu-Dumitrescu.

The conclusions are based on the following:

• A huge amount of work involved in carrying out microsurgical reconstructions of over 7,500 peripheral nerves in over 3,000 patients, 1,800 of which were nerve transplants for defects of peripheral nerves of the extremities, for posttraumatic brachial plexus paralyses (91), for replantations and/or revascularizations following partial or complete amputations of the extremities (24 out of which 23 successful) or for free transfers of functional composite tissues (53). For a more accurate picture of such an effort one should consider the operation time that these types of reconstruction involve: between 3 and 12 hours for each patient under general anaesthesia and for both the anaesthetist and the microsurgeon.

• Experimental microsurgery on rabbit ears

• The results of the histopathological examination of 500 postoperative neuromas of peripheral nerves repaired traditionally

• The Moberg test

• Pre, intra and postoperative monthly observations of the patients until their full recovery according to the criteria set by the International Reconstructive Microsurgery Society (postoperative intervals of 6-12-24 months)

• Taking pictures and recording pre, intra and postoperative stages

• The patients’ professional, social and familial reintegration

• The patients’ state of mind; level of cooperation

• Comparing results with those of classic and palliative repairs

• Comparing the data resulting from this experience with the information provided by the specialist literature of the world

• Completing the internationally defined reconstructive procedures with the personal ones, to produce a new concept

The conclusions drawn from my own experience can be summed up as follows: 

1. The fact that for hundreds of years posttraumatic paralyses of the spinal and partly cranial peripheral nerves have been considered insurmountable obstacles, is largely justified by a very limited concept of the restoration of neural “connections”. This implies:

• Little knowledge of the functional mechanism of the peripheral nerves and even less knowledge of the behaviour of the traumatized peripheral nerve 

• Little or no consideration of the importance of the extensive cerebral area assigned to the hand 

• No conceptualization of peripheral nerve reconstruction methods 

• Too frequent recourse to mechanical devices and operation procedures employing the muscles for some existing movement in a paralyzed territory so that the achieved movements have always been mistaken for recovery; no comprehension of the fact that the purpose should not be to simulate or train purely mechanical movements

• Although the research carried out throughout the world has been highly sophisticated over the past two decades, it has optimistically been focused on “a single cell”, organelle or growth factor and most often such efforts are focused on other methods than the “natural” ones of peripheral nerve reconstruction 

• Confidence in the evasive doctrines of degeneration-regeneration of the injured and reconstructed peripheral nerve has been tolerated for too long; conformity and passiveness have rendered the doubts cast on them unable to gain the strength and momentum to change the reconstructive concept

• The general principles of the reconstruction of soft extremity parts have been widely developed and extended over the past thirty years 

• Late division of surgery into distinct surgical specialties

• Slow development of magnifying devices and, consequently, tissue manipulation equipment 

• Even 40 years after magnifying devices were first used there is still confusion over what should be reconstructed, procedures are eagerly and carelessly applied and “planning” is limited to the microsurgical technique 

2. Reconstructive microsurgery (RM) is a new concept. Although the vision of reconstruction by neural transplant belongs to Philipeaux and Vulpian (1870), while Langhley and Hashimoto (1917) were the first to consider fascicular reconstruction, the new surgical procedures were actually introduced as late as 1968, when Bora and Millesi made that giant leap forward by creating worldwide receptiveness to the idea of soft part structure microsurgical reconstruction.

3. Thirty years of dissemination and improvement of the concept of microsurgical reconstruction and reconstructive microsurgical practice have led to feedback that changed a series of percepts but also raised many questions. Such developments are inevitable within and after real experience. The peripheral nerve microsurgical reconstruction concept is closely connected to the recovery process of severed and microsurgically reconstructed peripheral nerves.

4. Reconstructive microsurgery is a new concept, the concept of microsurgical reconstruction of tissues using specific means:

1. The operating microscope 

2. Microsurgical instruments; bipolar coagulator 

3. Special microsurgical suture material 

4. Microsurgical techniques specific to the types of tissue to be reconstructed

5. Specialized surgeons equipped for reconstructive microsurgery specific to that specialty

6. Special general anaesthesia conditions

7. Special operating room conditions 

8. Special intra and postoperative care conditions as well as the team trained and put together for this purpose.

5. Significant changes to peripheral nerve surgery were made primarily by the emergence of a new concept of nervous system repair. By introducing the microsurgical concept, surgeons have tried to improve the operating conditions and the visualization and manipulation of the delicate neural elements. In addition, they overcame the great obstacle in the way of successful peripheral nerve repair by introducing a new operating technique – the microsurgical reconstructive one. 

6. Classic epineural sutures are gradually abandoned in favour of fascicular sutures.

7. Of all the microsurgical peripheral nerve suture techniques, the fascicular group suture is currently the most appropriate for maximal recovery. Naturally, in adequate technical conditions and when “there is no alternative” the epiperineurial suture and especially the circumferential fascicular suture can prove useful. 

**Figure:**
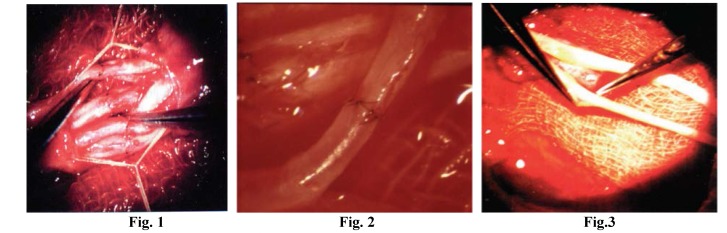
Fig. 1;Peripheral nerve microsurgical fascicular suture in fascicular groups In Fig. 1 see the removal of the nerve sheath, the epineurium, as well as the aligned, already sutured (coapted) fascicular groups. Fig. 2; In Fig. 2 the anastomosis of the main artery of the nerve, magnified 25 times. Fig. 3; Image exemplifies epineurial delimitation at the level of nerve graft segment extremities, its excision.

8. In 1982, associated anastomosis of the main artery of the nerve (0.2-1 mm in diameter) proved that in 70% of cases recovery was faster and better. The most difficult thing about this anastomosis is not the technical skill but finding the artery to which anastomosis can be performed.

Considering the major defect created by strokes as well as the data provided by the specialist literature describing a vascular system that is in excellent condition from an epiendoneural point of view, and the importance of this artery in embryonic life, in 1982 I decided to associate fascicular group reconstruction of the peripheral nerve with the anastomosis of the main artery of the peripheral nerve, i.e. the median nerve. There were two striking conclusions: 1. the recovery period was reduced to half and its quality doubled. 2. there was concern for the safety of the anastomosis carried out on a vessel 0.2-1 mm in diameter and the surrounding compressive fibrosis.

9. How old the lesion is, the patient’s age and the microsurgical concept are the key factors on which the level of recovery depends after a peripheral nerve lesion. Obviously, the shorter the posttraumatic period, the better the results. However, in young and very young patients, the age of the lesion does not represent a contraindication for grafting provided it is done for the purpose of gaining at least the tactile sensitivity.

There are several key reasons why I will not recommend laborious reconstruction during the emergency operation:

1. The injury is more or less contaminated, so, at this point, it is more exposed to infection than at the moment of the secondary operation.

2. The emergency procedures are basically meant to provide first aid treatment. In general, one has to be extremely lucky to come across a highly skilled reconstructive plastic surgeon who happens to be on regular duty. Iatrogenic damage (something that apparently can never be checked retrospectively), added to the initial injury, will complicate and diminish the actual chance of recovery.

3. For the time being, the emergency exploration cannot detect the extent of damage to the neural tissue of the peripheral nerve; while at the next stage, 3 weeks later, the length of the neuroma is clearly “outlined” and the microsurgeon alone has to perform appropriate, highly specialized surgery.

4. Provided I had the authority to do so, I would set up special regulations to be followed when performing emergency operations.

5. There are exceptions: when, from the very beginning, the patient is taken care of by a specialized unit or if replantation is involved, an operation that can be successfully carried out (i.e. with optimal motor and sensory results) only by a highly skilled and experienced specialist; of course, emergency reconstruction must have clearly defined goals: bone alignment, blood vessel (arteries and veins) repair, fixing the flexor and extensor tendons when this is possible without compromising reconstructed vessels and nerve anchoring, tegument suturing and immobilization will certainly be part of the program.

This may seem a simple enumeration of reconstruction stages and this may actually be the case when the surgeons involved understand what they are doing, the purpose of what they are doing and the fact that they did not learn their job by “hearsay” or “watching”. Reconstruction involves solid knowledge, and time-consuming study.

10. As for the level of the lesion, I could not notice any difference in the quality of the postoperative evolution of the peripheral nerves operated on within 6 months of the accident, regardless of the level of injury (hand, forearm, arm, foot, calf and thigh).

11. The “surgical bed”, the associated lesions as well as the number of surgical re-interventions (for example, the cases in which two nerves have been affected or major arteries have been severed) are matters that I find more important. In such cases the recovery period increases and the quality of the recovery is poorer. When the patient is young, when no more than 6 months have passed since the accident and when there were no previous surgical interventions, the quality of the repair can be as good as the one in the case of only one severed nerve.

12. The most difficult moment is adequate coaptation according to corresponding sensory and motor groups. The only conclusion at this point is that a rapid and safe intra-operative identification method would be really helpful; until then, relying on the “common sense” of sight and orientation under the operating microscope remains the only available option. 

13. As for the length of the nerve defect, the longest nerve defects covered by grafts measured 28 cm. I am not in the least convinced of the point held by many specialized studies that the longer a defect the poorer the postoperative results. To my surprise, the sensitivity level tests carried out on different lengths of defects within the first 30 days after the operation proved the presence of hypoesthesia, then of tactile sensitivity in 80% of the grafted median and posterior tibial nerves within a period of 7-30 days after the operation, as well as some degree of finger flexion in the high median nerve lesions or finger extension in external popliteal sciatic nerve lesions.

I will not question the tendency of the peripheral nerve to “grow” through its axonal buds especially if the proximal stump meets optimal requirements: clean section after delimiting the neuroma through excision. But the problem of the distal stump, of leading the nervous impulse from the proximal to the distal and vice versa plays, in my opinion, a major role in the quality of the restored fibre continuity. Under the present circumstances a good fascicular reconstruction is the most we can hope to achieve. When the peripheral nerve fibre becomes as important as the fascicle is now for reconstruction, I contend that the recovery will be almost “instantaneous”, taking no longer than a current neuropraxis. The fact that proximal regeneration is better than the distal one is due to the multiplication of the nerve fascicles and, moreover, of nerve fibres, a multiplication that is greater distally, resulting in higher stakes of coaptation and a greater difficulty in properly and technically carrying it out according to the standards from 2005. The problem of coaptation in fewer and “coarser” structures such as the ones at brachial plexus or arm level has been largely solved, but in the matter of “perfect” distal coaptation we are still some way behind. Special means (operating microscope, instruments, suture materials) permitting, recovery will be, figuratively speaking, “instantaneous”, as it happens in neuropraxis.

This is how I explain why in a higher lesion, where there are 1-2 or very few fascicles, the result is, “say”, equivalent to 8mm a day. In reality things are much more “serious”, there may be many more millimetres a day, something I have observed all too often.

It’s been a long time since I first made the same remark concerning the length of grafted nerve defects as well as the level of such defects: at the plexus, brachial, or antebrachial plexus the nerve defects quite often reach over 20-25 cm in length. Bearing this in mind, what kind of explanation for regeneration can we produce for the current use of the truncal ulnar nerve as a nerve graft in serious and older than 6 months – 1 year brachial plexus lesions (i.e. involving all the components), and in adults over 40-55, resulting in regaining flexion 8-12-21 days after the operation?

**Figure:**
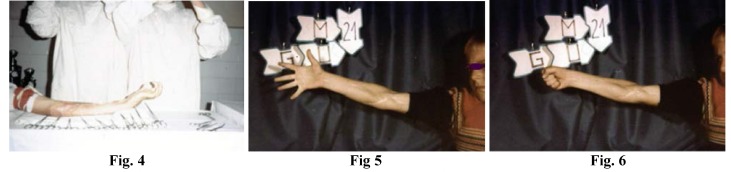
Fig. 4; Fig. 5; Fig. 6; The Volkmann Syndrome: Fig. 4. Extensive fibroses of muscles in two children. Surgery consisted in forearm fasciotomy, lengthening of deep flexor tendons, excision of m pronator teres, microsurgical reconstructions in two stages, at two and/or three months, of the median, ulnar nerves with nerve graft. Fig. 5, 6: Result at 21 months in the first child.

14. With respect to the location of the lesion and the length of the nerve defect, any time there is tension “to the limit” upon the future suture line, one must choose between suture under tension and nerve grafting. The specialist literature maintains that in 3 to 4 cm long defects the nerve graft should be taken into consideration. In reality, in median nerve lesions, the 2 to 3 cm long defects can be “compensated” by proximal and distal mobilization of the nerve. Things are different in the case of ulnar nerve defects in the proximal or median third of the forearm, where 1 cm long defects definitely need grafting.

In small 1-2.5 cm long ulnar nerve defects, I obtained better results after using the dorsal sensory branch of the ulnar nerve rather than a sural nerve segment as a donor nerve.

15. Drawing from Millesi’s understanding of the epinerve conjunctive tissue, since 1981 I have regarded the excision of the graft epinerve (at the graft extremities) as a reasonable and useful decision. It increased the chance of comfortable and high quality fascicular alignment during the operation and the postoperative reinnervation.

16. Nerve graft harvesting requires special attention and therefore all the grafts were harvested “by sight,” through an incision covering the whole length to be excised, without any traction – stretching (knowing that a 15% stretching compromises the nerve) so that the only conjunctive tissue surrounding the nerve would be the inner layer of the epinerve.

17. The 41 patients who underwent vascularized sural nerve graft and microvascular anastomosis are not enough to enable us to draw certain conclusions. That is why, the fact that 38 patients with vascularized grafts recovered exceptionally well may be purely coincidental. Nevertheless, the results achieved in such a short period of time are exceptional when compared with those obtained by common microsurgical grafting.

In 1985 I resorted to the international experience in using vascularized nerve grafts, being persuaded by the importance of adding vascularization to peripheral nerve reconstruction.

18. I do not believe in the effectiveness of the anterior ulnar nerve transposition in the cases indicated by specialist literature. Higher lesions have less chance of perfect recovery of the intrinsic musculature, but the big deep flexors can still recover. If transposition is carried out, the motor fascicles for the deep flexors are most often severed or stretched; in other words they are pointlessly sacrificed. A nerve graft is therefore indicated and preferred.

19. In case of "continuity lesions" or peripheral nerve lesion of the 6th degree, the diagnosis during the operation is necessary although it is very difficult to accurately identify the site of the lesion, the number of affected fascicles and the edges of the lesion. These data can be obtained as a result of a painstaking, irreproachable internal neurolysis and thus, reconstructive surgical intervention can proceed. The annoying, “endless” continuity lesions can however be carefully delimited. The absence of vascularization on a portion of the nerve affected by this kind of lesion, and the conjunctive tissue of the epinerve with its interfascicular extensions become a valuable guide for identifying the “limits” of this type of lesion. By feeling the nerve segment and permanently comparing it with the rest of the peripheral nerve, the surgeon may find signs of empty tube, more fibrous, more sclerotic content. Most frequently, if not always, the “continuity lesions” need healthy tissue excision and grafting of the initial nerve defect. The neurolysis alone is not able to sustain recovery of the injured peripheral nerve territory.

20. In cases of median and/or ulnar nerve paralyses, severe sequelas such as a neglected Volkmann, or severe, prolonged nerve ischemia, spectacular results can be achieved following fasciotomy and the restoring of nervous continuity even by using nerve grafts, but only if the deep flexor muscular mass still has “survival” chances.

21. In lesions due to nerve stretching, we believe that neurolysis “alone” is useless, the only real opportunity to recover being provided by healthy tissue excision and covering the defect by nerve graft.

22. Daily monitoring of the operated patient up to the 12th day for direct coaptation and the 21st day for indirect coaptation (nerve transplants) proved the presence of hypoesthesia and tactile sensitivity in the third, seventh and fourteenth day after the operation, on previously inert areas. They are clearly outlined on the 21st day following the operation. Timid flexion or extension movements and even incomplete and inaccurate flexions and extensions were evidenced during the same interval. Peripheral and less than peripheral movements were also evidenced in the 15 to 30-day interval following microsurgical reconstruction by long nerve transplants in complete brachial plexus paralyses and therefore reconstructions of “four out of five nerves”. These observations do not confirm the current physiopathological explanations provided by the international specialist literature on nerve recovery. I am certain that new theories on the recovery or so-called “regeneration” of the operated posttraumatic peripheral nerve will soon become available.

In 1980-1981, I observed the presence of the Tinel sign in the distal finger extremity after fascicular group reconstruction of the peripheral nerve 3-5-7 days after the operation. In 1982-1983, anxious that I could not find the Tinel sign in the same location within the same time lapse, at the patients’ insistence that they “could feel” the previously insensitive part, I started to closely monitor them for I could not believe them, since the peripheral nerve degeneration – regeneration theory said otherwise. After one year, I was positive that in 3-4-7 days, several disparaged hypoesthesia areas did appear on the initially denervated area and that by the 21st day they had extended over the entire previously denervated surface. In the absence of an objective factor I began to require the patient to very carefully attempt some basic movements that had been abolished before the operation. I thus realized that the movements frequently appeared 6-8-21 days following the operation. As I gained microsurgical experience, such movements began to appear as a matter of course in patients who met the essential conditions for good recovery: time lapse since the accident, younger than 45, fascicular group reconstruction.

23. I think somehow the posttraumatic peripheral nerve degeneration-regeneration theory has blocked our minds and we ignore one simple fact: any major trauma produces disorder of which the conjunctive tissue takes advantage, at the expense of noble tissues. When the skin is cut and no reconstruction succeeds, when our organs are badly traumatized and injured and there is no reconstruction afterwards or if the natural tissue order is not restored is there anything to prevent cell destruction and to help the body part resume its function? The same happens when a neuroma is formed proximally and distally by means of an oedema, disorientation, stupor, loss of direction and continuity, to which the conjunctive tissue is added to drive the last nail into the coffin, increasing the reaction to trauma of the peripheral nerve fascicles-fibres.

The neuroma is not the result of degeneration, degeneration being the result of trauma and conjunctive tissue invasion. This mechanism reduces the fascicle and endoneurial tube diameters resulting in the lack of normal supply of nutrients, by deficit to the proximal stump, and by absence to the distal one. Why is it that following secondary coaptation in due time, i.e. after continuity was resumed, degeneration and regeneration phenomena fail to follow the same pattern? Were they not severed and then sutured?! Deeper understanding of the phenomena and more adequate surgical means to suit the new level of understanding will create the opportunity for more rapid and higher quality recovery. Undoubtedly, in the future, when the peripheral nerve fibre suture becomes possible or genetic engineering takes over, the results will no longer have to be improved.

Why cling on the 8mm/day regeneration? Twenty years ago, when I completely believed in the 1-3 mm/day of peripheral nerve regeneration, I realized that this rate was not at all real. 

As long as a peripheral nerve can recover 90% following microsurgical reconstruction with or without nerve grafting, if the time lapse since the accident was optimal, if the patient is of the right age, if the reconstructive indication is adequate and if the microsurgeon does his/her best, why believe that a much better reconstruction, at the fibre level for example, or maybe with more accurate means, must fall under the “theory” instead of changing the theory by updating it to suit the needs of today and especially, those of the future?

24. None of the patients who underwent peripheral nerve microsurgical transplant on peripheral nerve defects needed palliative surgery.

25 None of the more than 3,000 patients microsurgically operated on peripheral nerves displayed any suppurating complication. During the operation or within the first three hours after the operation, four patients suffered ruptures along the suture line of the peripheral nerve; the suture was redone 24 hours later.

26. Limb replantations have a special status; there is a very strict “order” of carrying out the surgical intervention, an order set forth by the most outstanding microsurgeons in the world, which proves reasonable and relatively “simple”. Yet, I would make one observation: I only performed a small number of replantations (24, 23 of which successful) and I may not be entitled to such a remark, but my experience with peripheral nerve surgery leads me to believe that in the case of replantation operations the emergency intervention should be restricted to the simple nerve anchoring. Nerve continuity should be restored in a secondary intervention, quietly, under less pressure; 

Although the solution does not follow the recommendations of specialist literature, which maintains that peripheral nerve continuity be restored during the emergency intervention, I recommend anchoring as the most effective solution for the time being.

Here are the arguments in favor of this option:

1) it is impossible to determine the total length of the peripheral nerve lesion, the essential condition for a maximal result;

2) prolonged exposure of the fresh arterial and venous anastomoses can compromise replantation;

3) the already long duration of general anesthesia – usually from 6 to 12 hours – may lead to vital, unforeseen complications; such cases usually refer to patients with unknown medical history; 

Replantation is out of the question in a room without adequate personnel and equipment (microsurgeon, operating microscope, microsurgical instruments, and proper conditions for longer general anesthesia: 6-8-10-12 hours).

**Figure:**
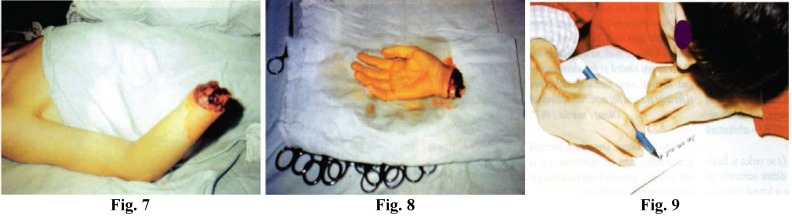
Fig. 7; Fig. 8; Fig. 9; Replantation case

27. Concerning brachial plexus reconstructions of posttraumatic paralyses I have reached the following additional conclusions:

The need to bypass the fibrosclerotic formation to avoid accidental rupture of the vessels with extremely fragile walls traumatized during the accident. 

The length of defects along the root-trunk-fascicle-nerve pathway is not relevant as long as there are adequate nerve graft sources 

It is preferable to use the truncal nerve grafts at least for connections with the nerves that have priority 

No matter how dim their prospects appear to be, the remaining roots should never be left out 

In most cases of brachial plexus paralyses due to avulsion-pulling out-stretching vascular damage is equally severe; therefore, I believe it is extremely useful and advisable to perform routine antebrachial fasciotomy during plexus exploration and reconstruction. 

**Figure:**
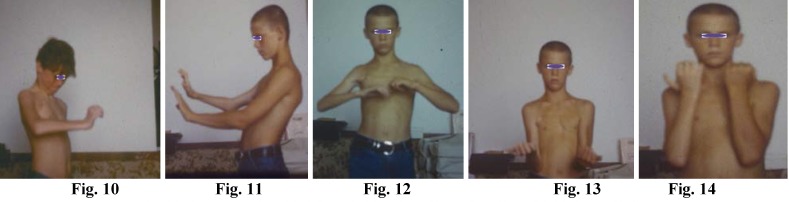
Fig. 10; Fig. 11; Fig. 12; Fig. 13; Fig. 14 Complete Brachial Plexus Palsy

Reconstruction of the completely paralized brachial plexus, is performed by sural nerve grafts, 6-12cm. long, between the medial, lateral fascicles to the median, musculocutaneous, ulnar nerves as well as the neurolysis of the posterior fascicle (radial and axilar nerves). Two months after reconstruction the patient can perform useful extension of fingers and hand (Fig. 20). Six months after surgery, the patient can perform complete extension of fingers and hand Fig. 21, the efficient flexion of the fingers Fig. 22, flexion of the forearm to the arm Fig. 23, supination Fig. 24. 

28. Special conclusions on functional free flap transfer:

Free microsurgical muscular transfer for the purpose of hand motor function recovery can only be carried out if certain requirements concerning the host area are met, such as:

1. Very good sensitivity on the territory of the palmary and digital median nerve 

2. Absence of any contractile muscles at the level of the forearm 

3. Existence of free joints at the metacarpophalangeal and interphalangeal levels 

4. The patient has will

**Figure:**
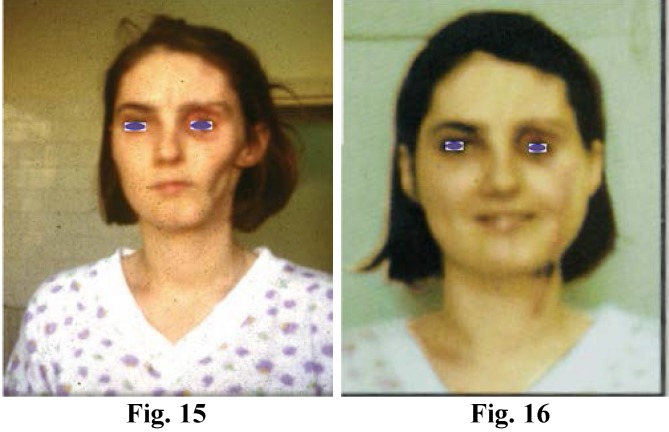
Fig. 15; Fig. 16; Progressive Facial Hemiatrophia. Reconstruction of Face Contour : the vessels of the latissimus dorsi free flap were anastomosed to the facialis vessels. One year later, the esthetic free flap modeling was performed (Fig. 15, Fig. 16)

Old facial paralysis treatment. To select the optimal donor nerve that can be used in the host area is often difficult in very old facial paralyses. In the best cases, such as facial paralyses after the excision of tumors, the free transfer of muscle together with nerves often makes the remaining facial nerve stump reusable. The function of the transferred muscle innervated by a former facial nerve is always superior to that of the muscle innervated by a hypoglossal or trigeminal nerve even if a nerve graft is inserted.

Combination with “Cross-Face” type nerve grafts for the purpose of facial reanimation: the reconstructive operation described by international specialist literature consists of two stages separated by 6-10 months: The first stage involves the transportation of the cross-face nerve graft, the second one focuses on providing its connection with the muscle nerve that is subject to free vascularized transfer. 

**Figure:**
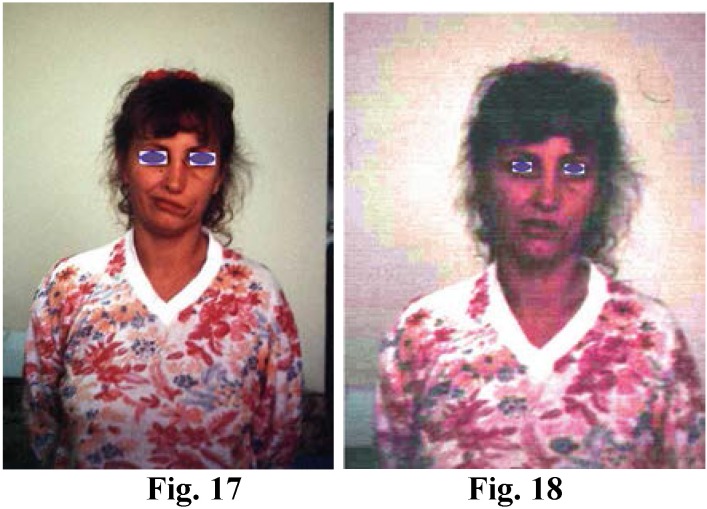
Fig. 17; Fig. 18; Old facial nerve paralysis: The one stage surgery, free flap transfer of serratus anterior muscle inervated with sural nerve graft 12-22 days after the operation, the patient displayed obvious contractions of the affected mouth commissure and upper eyelid

The following considerations influenced my decision to join the two stages into one and the same operation:

1. My experience and observations concerning peripheral nerves.

2. When the gracilis muscle is subject to a free transfer for covering an antebrachial muscular defect, there is no need for postponing nerve repair;

3. “The size” of the host nerve (mandible, zygomatic branch). It is hard to believe that in such delicate facial dissections, upon delimitation of the neural and vascular elements, fibroses will not appear around them, thus compromising at least part of these valuable elements. Why then have a second stage and inevitably further produce fibrosis since element delimitation becomes even more difficult? It is a pity that, once delimited, the nerve fibres, which are invisible to the naked eye, be deprived of continuity before postoperative fibrosis starts to affect them.

4. The local conditions of the face, with its entire vascularization, determine the rapid destruction of the non-innervated nerve graft. In this case, the motor plate cannot act as a stimulus to innervation recovery as the nerve graft lives through its function or the beginning of its impulse leading function.

5. If in cases of replantation or revascularization I am in favour of a secondary nerve repair operation, in this case, I believe that primary repair is crucial for the quality of the free muscular transfer; without it the free transfer is useless. I am thinking of the delicate function of the facial expressiveness, control of the mouth commissure and the opening-closing of eyelids. 

The one stage surgery that I propose consists of the following operation steps:

1. Delimiting the mandible branch and, if possible, the zygomatic component of the unaffected facial nerve;

2. Harvesting the sural nerve graft without pulling it out;

3. Indirect coaptation of the nerve graft to the unaffected end to the mandible branch of the facial nerve (and, possibly, to the zygomatic component) as well as nerve tunnelling strictly beneath the skin, usually on the upper and/or lower lip and bringing the remaining free end of the graft to the affected side of the face.

4. Harvesting the anterior serratus muscle (the last three branches) with the corresponding neurovascular pedicle.

5. Free transfer and positioning of the anterior serratus digitations at the level of the affected side of the face, ”spreading” and fixing the muscular digitations to the mouth commissure, the upper and lower eyelids.

**Figure:**
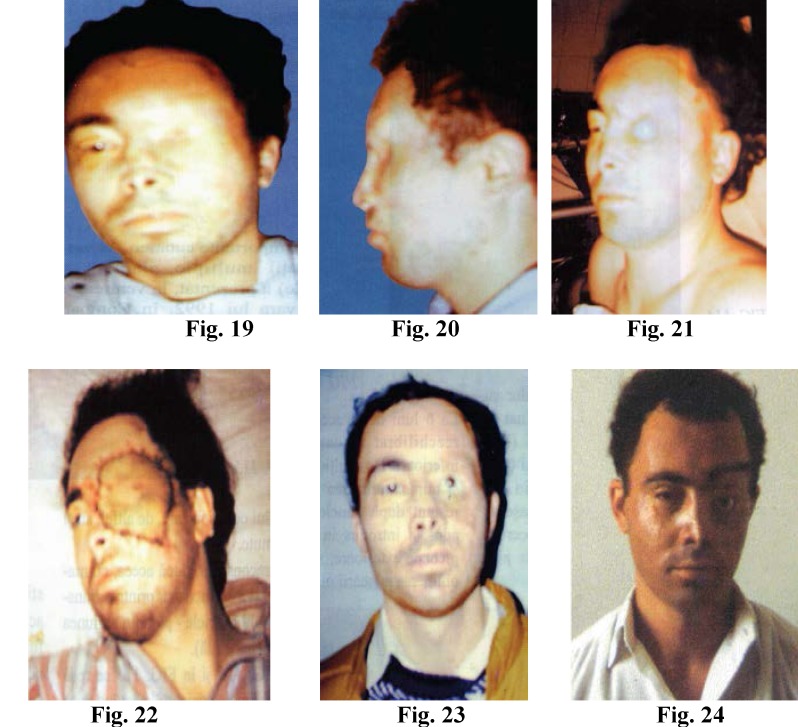
Fig. 14; Fig. 15; Fig. 16; Fig. 17; Fig. 18; Fig. 19; Eye-socket reconstruction, mobile upper eyelid, lower eyelid, eyebrow

29. My microsurgical experimental studies on the reconstruction of the rabbit ear nerves proved the existence of fibre continuity in all the histopathological examinations performed. The rabbit’s ear contains extremely fine nerves, with no more than 2-4 fibres.

30. The histopathological examinations performed on the 500 neuromas of the classic sutures on large nerves such as the median, ulnar and radial ones constantly revealed the absence of fascicular continuity. Only a tiny fraction of neuromas displayed some partial level of fibre continuity, the rest of the section being filled with classic suture threads granulomas within the conjunctive tissue.

31. The histopathological examinations carried out on the experimental vascular anastomoses of the rabbit’s ear (over 100 arteries and veins 0.2-1 mm in diameter) between1980-1985 revealed a 90.2% success rate.

32. Reconstructive microsurgery cannot be successfully performed in haste. Any seemingly minor mistake will compromise the entire 3-6-12 hour work.

33. Peripheral nerve reconstructive microsurgery complementary treatment:

The limb segment that underwent operation for reconstruction purposes is maintained in an elevated resting position. This means that:

A peripheral nerve reconstruction of the direct coaptation type at the hand-forearm level needs segment resting and immobilization in plaster splints over a 22-day period, then immobilization ends but another 30 days for one nerve and 45 days for two reconstructed nerves are needed before starting practicing passive and active movements; for six months physical effort is not recommended, and that includes lifting weights over 1-2 kg.

A peripheral nerve reconstruction of the indirect coaptation type located in the same region follows the same indication but the patient needs additional sick leave days and cannot start practicing hand/forearm movement within 45 days since the operation. It also involves special care for the donor area to ensure physiological healing.

A brachial peripheral nerve or nerves reconstruction by direct or indirect coaptation needs a 30-day period of bed rest immobilization in order to avoid the weight of the limb segment; the same recommendations apply here.

Cranial-facial peripheral nerve reconstructions usually need a 14-21-day period of “immobilization”, which is actually meant to avoid tension and oedema in the region; the patient can return to work if the operated area is protected from cold or manoeuvring, but the doctor may recommend an additional 14 day sick leave.

Pelvic extremity peripheral nerve reconstructions require total bed rest in the recommended position so as not to produce tension on the reconstruction line or lines; this compulsory 30-day immobilization period is followed by another 2 months (which are part of the sick leave) when, for six weeks, the patient is allowed to walk without touching the ground with the operated limb and then to walk with a cane for the remaining two weeks.

Brachial plexus reconstructions involve bed rest immobilization in a pseudo-Dessault, horizontal position, avoiding tensions between the neck-cervical region and the operated area, preventing the thoracic extremity from exerting pressure through its weight, without the least amount of tension on the usually multiple reconstruction lines; movement from the logical positions is forbidden in order to preserve the chances for the best possible results. If the patient’s job provides little guarantee for the safety of joint mobilization to maintain the allowed amplitude, it is safer and more advisable to immobilize the patient for a month than risk coping with an important deficit throughout the patient’s life. Usually, after such reconstructions, the patients need a six-twelve month sick leave to gather strength and energy and to concentrate on the exercises and workout they need to do.

All the recommendations in the case of hand-forearm grafting apply to peripheral nerve reconstruction upon replantation.

After the end of the compulsory immobilization periods, i.e. the intervals following the 22 days – 1 month period after the operation, the passive and active gradual mobilization of the reconstructed segment components must begin. The idea is that this mobilization must be performed by the patient from the very beginning, even if the patient is a child, since the patient’s mental cooperation is indispensable after this type of reconstruction. The movement will help the patient regain the kind of mobility any patient wants, that is to regain the normal movement abilities before the accident, with the obvious limitations in each patient’s case, but always comparing it with the function of the healthy, non-traumatized opposite limb segment. Cooperation and results will completely depend on such “details”.

34. Diapulse-therapy associated with surgery facilitates axonal growing and breaking of the barrier at the suture level. This is proved by the presence of the Tinel sign between the metacarpophalangeal joint (MPJ) and the third phalanx (F3) during the first three days following the operation in all the patients who underwent fascicular suture associated with Diapulse therapy (DPT).

In the 1980s, in the Plastic Surgery Clinic, Professor Agrippa Ionescu had a lot of experience concerning the role of the electromagnetic field in treating burns. His conclusions such as reduction of the oedema, cell membrane repolarization, became arguments in favour of using Diapulse therapy as a complementary treatment to be employed immediately after microsurgical reconstruction. The ultimate conclusion was that every time DPT treatment was associated for 12-21 days with fascicular group reconstructions, the period in which the Tinel sign emerged dropped from 3-7 days to 2-3 days.

We started from the following assumptions:

1) DPT increases blood flow, enhances circulation and, therefore, raises the oxygen level in the tissues;

2) In case of circulatory disorder, which is always present in the posttraumatic – postoperative period, by restoring the electric potential of cells, DPT also restores their function, reduces circulatory disorder, causes oedema to shrink or disappear along with all its negative consequences, such as distension, pain, the emergence of scar tissue.

35. It is vital that the results be assessed for groups of patients operated by one and the same surgeon-microsurgeon. Apparently the same technique, applied to apparently the same category of patients, can yield opposite results or just minimal improvement in comparison with results following conventional surgery. At present, as a result of microsurgical interventions, in the best of cases (within a 6-month period after the accident in patients younger than 45), recovery can reach 75-90%. These are the real parameters of authentic reconstructive microsurgery. We should never encourage microsurgeons to achieve results that only show some improvement in comparison with the results yielded by classic techniques and be content with the patient’s satisfaction with such minimal improvements. Patients may be happy even with a 10-20% improvement, but they are not in a position to estimate their real chance of “maximal” recovery. The surgeon alone can justly appreciate what was achieved and what might have been achieved. Therefore high quality microsurgery can only be performed by doctors with a high level of professional conscience.

36. It is important that these microsurgical procedures be constantly evaluated and openly compared with conventional methods not only from a functional and aesthetic point of view, but also taking their actual cost into account. Microsurgery has the potential of reducing the number of hospital days, the period of functional handicap and, more importantly, it provides good prospects for total, or almost total, functional recovery. It is clear that over the next decade microsurgeons must include social considerations alongside medical ones when they evaluate their work. 

## References

[1] Terzis Julia K (1987). Microreconstruction of Nerve Injuries.

[2] Terzis Julia, Smith Kevin L (1990). The Peripheral Nerve Structure, Function and Reconstruction.

[3] Terzis Julia K (1987). Microreconstruction of Nerve Injuries.

[4] Rollin Daniel, Julia K. Terzis (1977). Reconstructive Microsurgery.

[5] Jewett Don L, McCarroll H. Relton (1980). Nerve Repair and Regeneration.

[6] Buncke Harry J (1991). Microsurgery: Transplantation-Replantation,.

[7] King Maurice, Bewes Peter (1987). Trauma.

[8] Harii Kiyonori (1983). Microvascular Tissue Transfer-Fundamental Techniques and Clinical Applications.

[9] Mathes Stephen J, Stephen J Foad (1982). Clinical Applications for Muscle and Musculocutaneous Flaps.

[10] Jurkiewicz M. J., Krizek Thomas J, Mathes Stephen J, Aryan Stphen (1990). Plastic Surgery.

[11] Strauch Berish, Vasconez Luis O, Hall-Findlay Elizabeth J (1990). Grabb’s Encyclopedia of Flaps.

[12] John Bostwick III (2000). Plastic and Reconstructive Brest Surgery.

[13] Georgiade G. S., Riefkohl R, Levin Scott.L (1997). Plastic, Maxillofacial and Reconstructive Surgery.

[14] Aston Sherrell. J, Beasley Robert W, Thorne Charles H. M (1997). Grabb and Smith’s Plastic Surgery.

[15] Denischi A., E. Proca, Doina Ionescu (1988). vol. III – Ortopedia / Tratat de patologie chirurgicala.

[16] Green David P (1988). Operative Hand Surgery.

[17] Ionescu Doina (1989). Microchirurgia nervilor periferici.

[18] Ionescu Doina (1995). Microchirurgia reconstructive in Romania.

[19] Dumitrescu-Ionescu Doina (1999). Microchirurgia Reconstructiva.

[20] Porth Mattson Carol (1990). Pathophysiology- Concepts of Altered Health States.

[21] Kandel Eric R, Schwartz James H, Jessell Thomas M (2000). Principles of Neural Science.

[22] Adams Raymond D, Victor Maurice (1994). Principles of neurology.

[23] Lindsay Kenneth W, Bone Ian (2001). Neurology and Neurosurgery Illustrated.

[24] Silbernagl Stefan, Lang Florian (2000). Color Atlas of Pathophysiology.

[25] Fuller Geraint (2004). Neurological Examination Made Easy.

[26] Arslan Orhan (2001). Neuroanatomical basis of Clinical Neurology.

[27] Snell Richard S (2001). Clinical Neuroanatomy.

[28] Atwood H. L, Mc Kay W. A (1989). Essentials of Neurophysiology.

[29] Wilson-Pauwels Linda, Akersson J. Elizabeth, Steward A Patricia (1988). Cranial Nerves, Anatomy and Clinical Comments.

[30] Berger A. Richard, Weiss Arnold-Peter C (2004). Hand Surgery.

[31] Weisberg Leon A, Strub Richard L, Garcia Carlos A (1987). Decision Making in Adult Neurology.

[32] Converse John Marquise Reconstructive Plastic Surgery.

[33] Sabin TD, Swift TR, Jacobson RR (1993). Leprosy. In: Dyck PJ, Thomas PK, eds. Peripheral neuropathy.

[34] Bosch EP, Mitsumoto H (1991). Disorders of peripheral nerves. In: Bradley WG, Daroff RB, Fenichel GM, Marsden CD, eds. Neurology in clinical practice.

[35] Stewart JD (1993). Focal peripheral neuropathies.

[36] Bracker MD, Ralph LP (1995 ). The numb arm and hand. Am Fam Physician.

[37] Thomas PK, Ochoa J (1993). Symptomatology and differential diagnosis of peripheral neuropathy. In: Dyck PJ, Thomas PK, eds. Peripheral neuropathy.

[38] (1983-1999). Clinics in Plastic Surgery.

[39] (2002-2005). Clinics in Plastic Surgery.

[40] (1990-1999). Plastic and Reconstructive Surgery.

[41] Yoon Ho Lee, Hyun Chul Kim, Jae Seung Lee, Weon Jin Park (1999 ). Surgical Reconstruction of the Contracted Orbit. Plastic and Reconstructive Surgery.

[42] Fu-Chan Wei, Ferit Demirkan, Hung-Chi Chen, I-How Chen, Chun-Ta Liao, Sheng-Po Hau (1999 ). Management of Secundary Soft Tissues Deficits following Microsurgical Head and Neck Reconstruction by Means of Another Free Flap. Plastic and Reconstructive Surgery.

[43] Kazuki Ueda, Kiyonori Harii, Atsushi Yamada (1994 ). Free Neurovascular Muscle Transplatation for the Treatment of Facial Pralysis Using the Hypoglossal Nerve as a Recipient Motor Surce. Plastic and Reconstructive Surgery.

[44] J. Henk Coert, A. Lee Dellon (1994 ). Clinical Implications of the Surgical Anatomy of the Sural Nerve. Plastic and Reconstructive Surgery.

[45] Kazuya Matsumoto, Hideki Nakanishi, Yoshio Urano, Hiroaki Nagae (1999 ). Lower Eylid Reconstruction with a Cheek Flap Supported by Fascia Lata. Plastic and Reconstructive Surgery.

[46] Fuminori Kanaya, John Firrell, Tsu-Min Tsai, Warren C Breidenbach (1992 ). Functional Results of Vascularized versus Nonvascularized Nerve Grafting. Plastic and Reconstructive Surgery.

[47] Gurhan Ozcan, Saleh Shenaq, Batool Mirabi, Melvin Spira (1993 ). Nerve Regeneration in a Bony Bed: Vascularized versus Nonvascularized Nerve Grafts. Plastic and Reconstructive Surgery.

[48] Kazuki Ueda, Kiyonori Harii, Atsushi Yamada (1994 ). Long Term Follow Up of Nerve Conduction Velocity in Cross FACE Nerve Grafting for the Treatment of Facial Paralysis. Plastic and Reconstructive Surgery.

[49] Lucie Capek, Howard M/ Clarke, Cristine G. Curtis (1998 ). Neuroma-in Continuity Resection: Early Outcome in Obstetrical Brachial Plexus Palsy. Plastic and Reconstructive Surgery.

[50] Yuhei Yamamoto, Hidehiko Minakawa, Tsuneki Sugihara, Yoshihisa Shintomi, Kunihiko Nohira, Tetsunori Yoshida, Hiroharu Igawa, Takehiko Ohura (1994 ). Facial Reconstruction with Free-Tissue Transfer. Plastic and Reconstructive Surgery.

[51] Georgio C. La Scala, Sean B. Rice, Howard M. Clarke (2003 ). Complication of Microsurgical Reconstruction of Obstetrical Brachial Plexux Palsy. Plastic and Reconstructive Surgery, Lippincott Williams&Wilkins.

[52] Perry J. Johnson, Anu Bajaj-Luthra, Ramon Llull, Peter Johnson (1997 ). Quantitative Facial Motion Analysis after Functional Free Muscle Reanimation Procedures. Plastic and Reconstructive Surgery.

[53] Muzzafer Altintas, Yagmur Aydin, Akin Yucel (1998 ). Eye Socket Reconstruction with the Prefabricated Temporal Island Flap. Plastic and Reconstructive Surgery.

[54] Kiyonori Harii, Hirotaka Asato, Kotaro Yoshimura, Yasushi Sugawara, Takashi Nakatsuka, Kazuki Ueda (1998 ). One Stage Transfer of Latissimus Dorsi Muscle for Reanimation of a Paralysed Face: A New Alternative. Plastic and Reconstructive Surgery.

[55] Tad R. Heinz, Patricia A. Cowper, Scott Levin (1999 ). Microsurgery Costs and Outcome. Plastic and Reconstructive Surgery.

[56] Akihico Takushima, Kiyonori Harii, Hirotaka Asato, Akira Momosawa (2005 ). Revisional Operation Improve Results of Neurovascular Free Muscle Transfer for Treatment of Facial Paralysis. Plastic and Reconstructive Surgery, Lippincott Williams&Wilkins.

[57] Roberto Adani, Luigi Trallo, Ignatio Marcoccio, Riccardo Cipriani, Chiara Gelati, Marco Innocenti (2005 ). Hand Reconstruction Using the Thin Anterolateral Thigh Flap. Plastic and Reconstructive Surgery, Lippincott Williams&Wilkins.

[58] Aleid C.J. Ruijs, Jean Bart Jaquet, Sandra Kalmijn, Steven E.R. Hovius (2005 ). Median and Ulnar Injuries: A Meta-Analysis of Predictors of Motor and Sensory Recovery after Modern Microsurgical Nerve Repair. Plastic and Reconstructive Surgery, Lippincott Williams&Wilkins.

[59] Ugrenovic Z. Sladijana, Jovanovic D. Ivan, Vasovic P. Lijiliana, Stefanovic J. Natalija, Kovacevic T. Predrag, Stojanovic R. Vesna (2005 ). Neurovascular Stalk on the Superficial Sural Flap: Human Fetus Anatomical Study. Plastic and Reconstructive Surgery, Lippincott Williams&Wilkins.

[60] Elliot H. Rose (2005 ). Autogenous Fascia Lata Grafts: Clinical Applications in Reanimation of the Totally or Partially Pralyzed Face. Plastic and Reconstructive Surgery, Lippincott Williams&Wilkins.

[61] David Chwei-Chin Chuang, Samir Mardini, Hae-Shya Ma (2005 ). Surgical Strategy for Infant Obstetrical Brachial Plexus Palsy: Experience at Chang Gung Memorial Hospital. Plastic and Reconstructive Surgery, Lippincott Williams&Wilkins.

[62] York J. Yates, Carlos L. Farias, Faeza R. Kazmier, Charles L. Puckett, Matthew J. Concannon (2005 ). Plastic and Reconstructive Surgery, Lippincott Williams&Wilkins.

[62.1] Neil W. Bulstrode, Douglas H. Harrison (2005 ). The Phenomenon of the Late Recovered Bell’s Palsy: Treatment Options to Improve Facial Symmetry. Plastic and Reconstructive Surgery, Lippincott Williams&Wilkins.

